# A quantitative approach for determining the role of geometrical constraints when shaping mesenchymal condensations

**DOI:** 10.1007/s10544-019-0390-0

**Published:** 2019-04-08

**Authors:** Valentina Onesto, William B. Barrell, Mary Okesola, Francesco Amato, Francesco Gentile, Karen J. Liu, Ciro Chiappini

**Affiliations:** 1 Department of Experimental and Clinical Medicine, University of Magna Graecia, 88100 Catanzaro, Italy; 2 Center for Advanced Biomaterials for HealthCare@CRIB, Istituto Italiano di Tecnologia, Largo Basanti e Matteucci 53, 80125 Naples, Italy; 3 Centre for Craniofacial and Regenerative Biology, Dental Institute, King’s College London, London, SE1 9RT UK; 4 Department of Electrical Engineering and Information Technology, University Federico II, Naples, Italy

**Keywords:** 3D *in vitro* models, Mesenchymal condensation, High-content imaging, Microtopography, Stem cells, Embryogenesis

## Abstract

In embryogenesis, mesenchymal condensation is a critical event during the formation of many organ systems, including cartilage and bone. During organ formation, mesenchymal cells aggregate and undergo compaction while activating developmental programmes. The final three-dimensional form of the organ, as well as cell fates, can be influenced by the size and shape of the forming condensation. This process is hypothesized to result from multiscale cell interactions within mesenchymal microenvironments; however, these are complex to investigate *in vivo*. Three-dimensional *in vitro* models that recapitulate key phenotypes can contribute to our understanding of the microenvironment interactions regulating this fundamental developmental process. Here we devise such models by using image analysis to guide the design of polydimethylsiloxane 3D microstructures as cell culture substrates. These microstructures establish geometrically constrained micromass cultures of mouse embryonic skeletal progenitor cells which influence the development of condensations. We first identify key phenotypes differentiating face and limb bud micromass cultures by linear discriminant analysis of the shape descriptors for condensation morphology, which are used to guide the rational design of a micropatterned polydimethylsiloxane substrate. High-content imaging analysis highlights that the geometry of the microenvironment affects the establishment and growth of condensations. Further, cells commit to establish condensations within the first 5 h; condensations reach their full size within 17 h; following which they increase cell density while maintaining size for at least 7 days. These findings elucidate the value of our model in dissecting key aspects of mesenchymal condensation development.

## Introduction

In the embryo, the formation of bone and cartilage is induced by a mesenchymal condensation process in which previously dispersed mesenchymal cells gather together into a tightly packed cell mass (Hall and Miyake 2000). The size and shape of the condensed cell mass define the final three-dimensional form of the tissue. Multiscale cellular interactions play a critical role in establishing the permissive microvenironment conditions to induce condensations, which initiate chondrogenic differentiation during embryonic development (Stott et al. 1999). Although advances in molecular biology have allowed a systematic investigation of the role of morphogens in regulating the differentiation and growth of chondrogenic cells and tissues (Ichinose et al. 2005; Niswander 2003; Daniels et al. 1996), the role of biophysical and extracellular matrix microenvironmental cues are less well understood.

It has been appreciated for nearly a century that skeletal morphogenesis involves interactions between cell intrinsic factors (e.g. genetic cues) and extrinsic factors including environmental constraints (Murray and Huxley 1925). Most parameters that regulate the reaction-diffusion of chemical factors and the mechanochemical interaction between cells and extracellular matrix on chondrogenic patterning (Oster et al. 1983) are based on *in vivo* observations. While theoretical models have highlighted their contributions, they have yet found little experimental validation (Glimm et al. 2012). Chondrogenic morphogenesis *in vivo* is a markedly three-dimensional process and the presence of surrounding tissues provides physical constraints to the cell population, which plays a role in the establishment of the condensation (Klumpers et al. 2014; Cukierman et al. 2001).

*In vitro* systems can provide a degree of experimental validation in a controlled environment; but their two-dimensional nature and the lack of scope to modulate biophysical interactions restricts the quality of information they can provide into the mechanisms of chondrogenic morphogenesis (Rottmar et al. 2010). A more three-dimensional system, such as the micromass model, can better mimic *in vivo* conditions and can also be used to test cell intrinsic and extrinsic parameters required for morphogenesis (Archer et al. 1985). This approach has since been used to determine the relative contributions of signaling factors and mechanical compression during differentiation of the tooth, where it has been shown that artificial compaction is sufficient to induce cell fate switches (Mammoto et al. 2011). 3D substrates for cell culture have been extensively used to achieve directional alignment or migration of mesenchymal cells (Rashidi et al. 2014), to study the role of mechanical stiffness (Fuhrer et al. 2013) and the influence of surface properties including charge (Webb et al. 1998) and wettability (Webb et al. 1998; Arima and Iwata 2007) on adhesion (Bacakova et al. 2011), phenotype and functionality of mesenchymal stem cells (Curran et al. 2005). Therefore, we set out to adapt our micromass cultures for use with our synthetic three-dimensional environments.

To understand what role geometric boundary conditions can play on condensation patterning, we used image analysis of mouse embryonic skeletal progenitor cells (ESPCs) in standard micromass culture to extract shape descriptors of condensation morphology, and used them to direct the design of 3D polydimethylsiloxane (PDMS) microenvironments. We identified the covalent binding of (3-aminopropyl)triethoxy silane (APTES) followed by fibronectin physisorption as the ideal strategy for long-term cell adhesion to PDMS, enabling the establishment of patterned 3D mesenchymal condensations for up to 7 days. High content live imaging analysis enabled determining the characteristic features of the early stages of condensation and to relate them to the degree of geometric constraining. In our system, ESPC condensations established within 5 h and grew in size up to 17 h, following which they increased in cell density up to 7 days. Uniaxial geometric constraint through microgrooves highlighted a groove-size dependent effect on condensation growth.

## Results

### Identifying key descriptors of condensation morphology

**Fig. 1:**
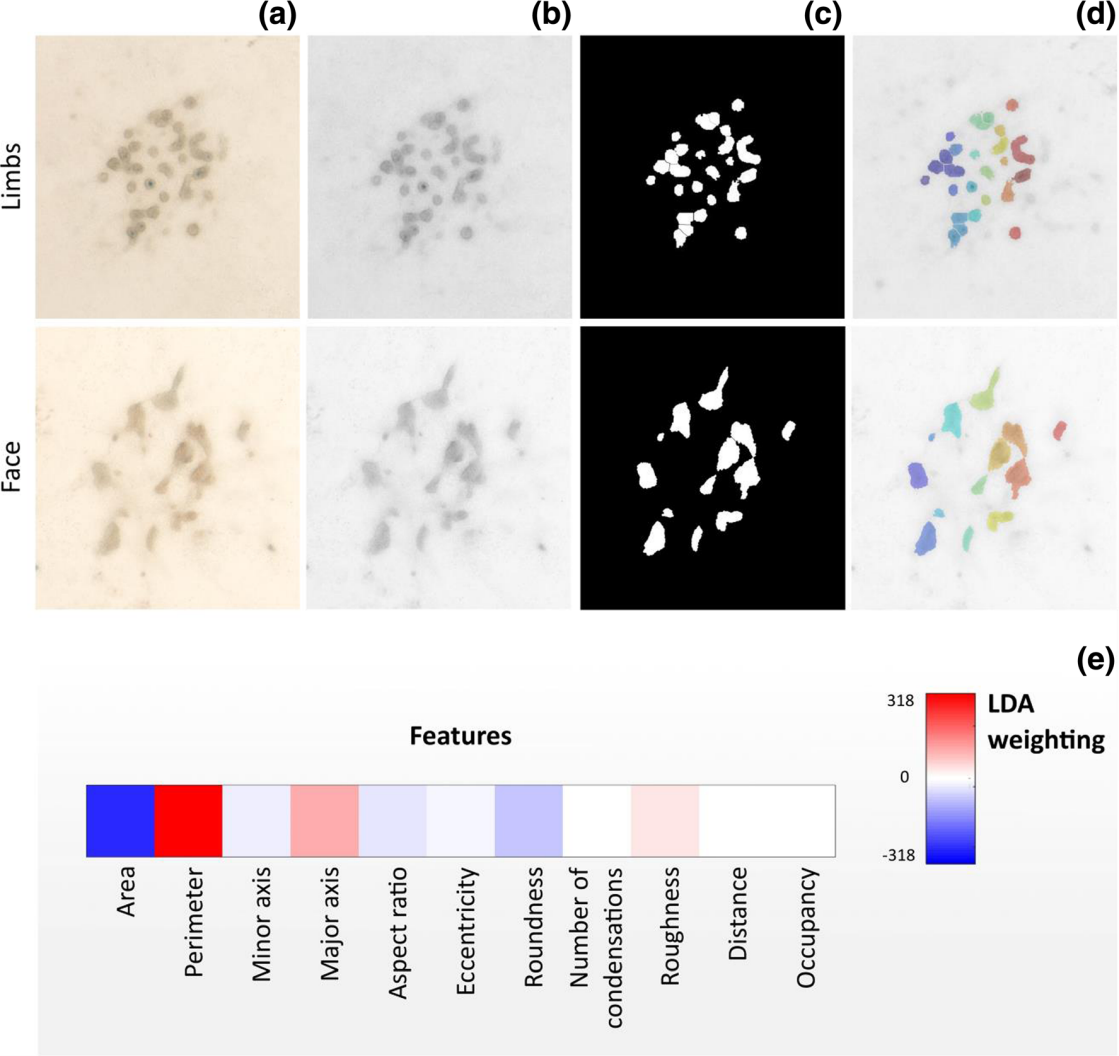
Identification of morphological features discriminating between limb buds and face condensations. (**a**) Brightfield stereoscopic images of condensations are (**b**) converted to grayscale, (**c**) binarized and (**d**) watershed segmented to identify individual objects. (**e**) Weighting coefficients for morphological descriptors of individual condensation arising from linear discriminant analysis between face and limb condensations. Higher absolute values indicate most significant descriptors

Condensation morphology is dependent on the local environment and represents an important large-scale manifestation of the global organizational and physiological state of cells across body sites. Comparing morphological information can provide guidelines when designing environmental cues that direct the establishment and development of condensations. To this end, we developed a Matlab® script for analysis of brightfield images (Fig. 1a). The script locates and isolates individual condensations (Fig. 1b-d) in micromass cultures of ESPCs isolated from the facial prominences and the limb buds. The analysis of the condensations extracted 11 individual shape descriptors used to characterize the micromass culture (Table 1). Linear discriminant analysis identified that *area, perimeter, major axis* and *roundness* were the most prominent morphological differences between face and limb condensations (Fig. 1e). In particular, limb bud condensations displayed more rounded and shorter shapes (i.e. lower aspect ratios) while facial condensations presented higher branching.

**Table 1:** List of shape descriptors and their definition

Property name	Description
Area	Actual number of pixels in the region
Perimeter	Distance around the boundary of the region
Eccentricity	Eccentricity of the ellipse that has the same second-moments as the region, returned as a scalar. The eccentricity is the ratio of the distance between the foci of the ellipse and its major axis length. The value is between 0 and 1. An ellipse whose eccentricity is 0 is actually a circle, while an ellipse whose eccentricity is 1 is a line segment
Minor axis	Length of the minor axis of the ellipse that has the same normalized second central moments as the region
Major axis	Length of the major axis of the ellipse that has the same normalized second central moments as the region.
Aspect Ratio	Major axis/minor axis ratio.
Roundness	$4\times \frac{\left[\mathrm{Area}\right]}{\uppi \times {\left[\mathrm{Major}\ \mathrm{axis}\right]}^2}$. A value of 1.0 indicates a perfect circle. As the value approaches 0.0, it indicates an increasingly elongated shape
Number of condensations	Number of regions in the image.
Roughness	$\frac{\left[\mathrm{Convex}\ \mathrm{Perimeter}\right]}{\left[\mathrm{Perimeter}\right]}$. It will take the value of 1 for a convex object, and will be less than 1 if the object is not convex. Objects having high roughness values show irregular boundaries.
Distance	Average of the 4 nearest-neighbor distance (calculated as centroid to centroid distance)
Occupancy	A circular region having as extremes of the diameter the most distant pixels belonging to the condensation is first selected. In this region the occupancy is given by the ratio between the area of the pixel belonging to the condensations and the total area.

### Establishing a three-dimensional model for ESPC culture

**Fig. 2:**
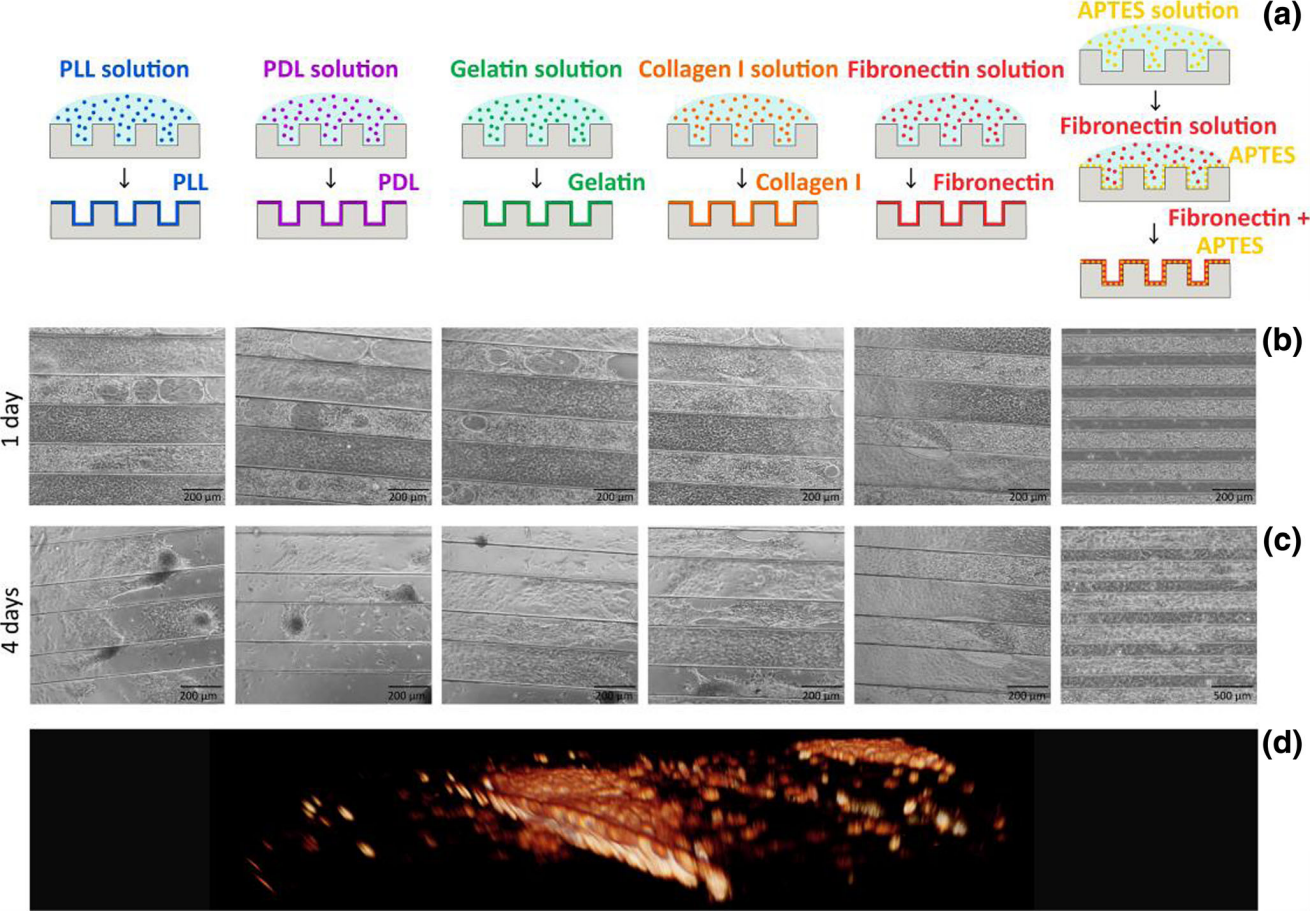
Establishment of microgroove culture system for ESPCs. **a** The PDMS substrates are functionalised with different coating strategies. **b**-**c** Phase-contrast images of ESPCs cultured on substrates with different coating strategies at (**b**) 1 DIV and (**c**) 4 DIV. Cell detachment and aggregation is visible at day 4 except for the APTES+fibronectin strategy. **d** Volumetric reconstruction of a confocal stack shows the 3D shape of the condensations in the microgrooves

Having identified that the *axis length* and *roundness* are significant contributors to site-specific variance in mesenchymal condensation, we employed microgrooves as an ad-hoc model to uniaxially constrain condensations, and modulate these key features *in vitro,* in order to dissect their role in skeletal shape generation. Thus, we generated PDMS microgrooves with 100 μm depth and variable width, selected at 25, 50, 100, 200 and 300 μm. To overcome the limited ability of PDMS to sustain cell adhesion and proliferation, we explored biofunctionalization strategies, including direct physisorption of poly-L-lysine (PLL), poly-D-lysine (PDL), gelatin, collagen I, fibronectin, and physisorption of fibronectin to a covalently bound (APTES) monolayer (Fig. 2a-c). It is known that the formation of a cationic coating layer on PDMS, despite the presence of positive charges at the cell interface, can improve the cytocompatibility of the surface in comparison to umodified PDMS (Nourmohammadi et al. 2015; Kuddannaya et al. 2013). The APTES + fibronectin surface modification approach outperformed the competing strategies in improving the attachment and proliferation of ESPCs. Fibronectin efficiently adsorbed on the APTES layer, enabling maintenance of ESPCs in culture for up to 7 days. In comparison, the alternative approaches exhibited significant detachment of embryonic mouse cells starting from day 3 in culture (Fig. 2c). The ESPCs cultured within this system assembled into three-dimensional cell bundles within each groove, establishing condensations therein (Fig. 2d).

### Developing a tool for high content imaging analysis of mesenchymal condensations

Quantifying key morphological features of the mesenchymal condensations within the 3D *in vitro* model is crucial to understanding the role of geometric constraints. High content live imaging analysis provides unprecedented power in dissecting the transition between cell seeding and the establishment of condensations. To this aim we developed a custom analytical tool capable of identifying individual condensations and track their development across a time-series, in order to determine the evolution of key shape descriptors.

**Fig. 3:**
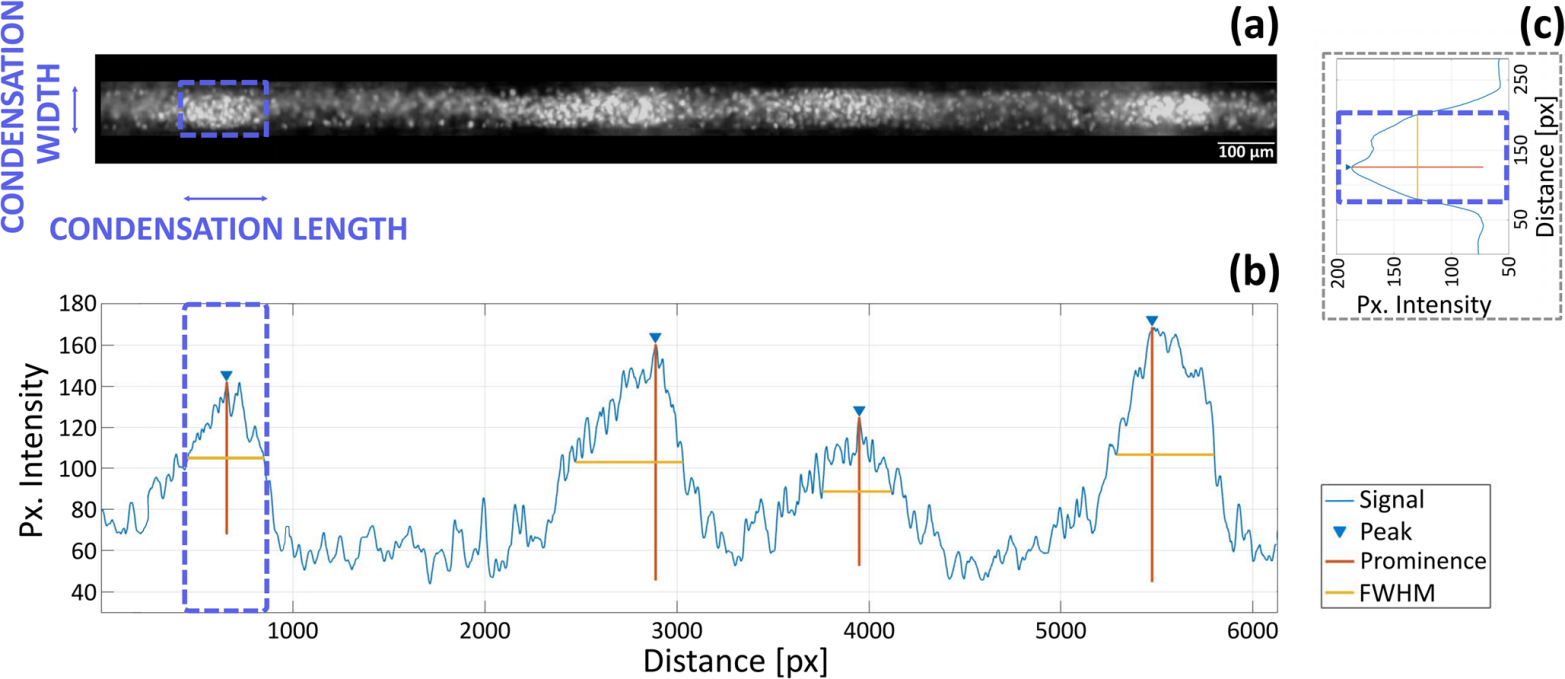
Strategy for the analysis of condensation morphology by high content live imaging. **a** Individual grooves are extracted from the image. **b** Plot of the fluorescence intensity along the minor axis of the groove for the highlighted condensation. Fluorescence intensity is averaged over the major axis of the groove within the blue box. **c** Plot of the fluorescence intensity along the major axis of the groove. Fluorescence intensity is averaged over the minor axis of the groove. The light and dark bands in the fluorescence image correspond to peaks and valley in the graph, respectively. Individual condensations are identified by peaks in the plot. Their length is defined by the FWHM of the peak

The script isolates individual grooves throughout a time series (Fig. 3a). Within each groove it identifies condensations at the final timepoint and tracks them backwards to their initial establishment. The condensations are detected by the local increase in cell density observed through the increased fluorescence intensity of Hoechst live nuclear staining. The script averages Hoechst intensity along the minor axis of the groove, to generate an intensity line plot along the major axis (Fig. 3b). Condensations are defined by the peaks identified in this plot. Their length, defined as their size along the major axis of the groove, is determined by the full width at half maximum of the associated peak (FWHM). For each peak/condensation identified, their width is given by the FWHM of Hoechst fluorescence averaged along the major axis of the groove for the length of the condensation (Fig. 3c). Additionally, further shape descriptors for the condensations (Table 1) can be derived from this data. These tools can track the number and morphology of condensation across time-lapse live imaging experiments as well as compare these parameters across endpoint analysis on fixed samples. This enables close investigation of the initial steps of condensation by live imaging, while tracking their further development with selected time-point analysis.

### Influence of geometric constrains on mesenchymal condensations

**Fig. 4:**
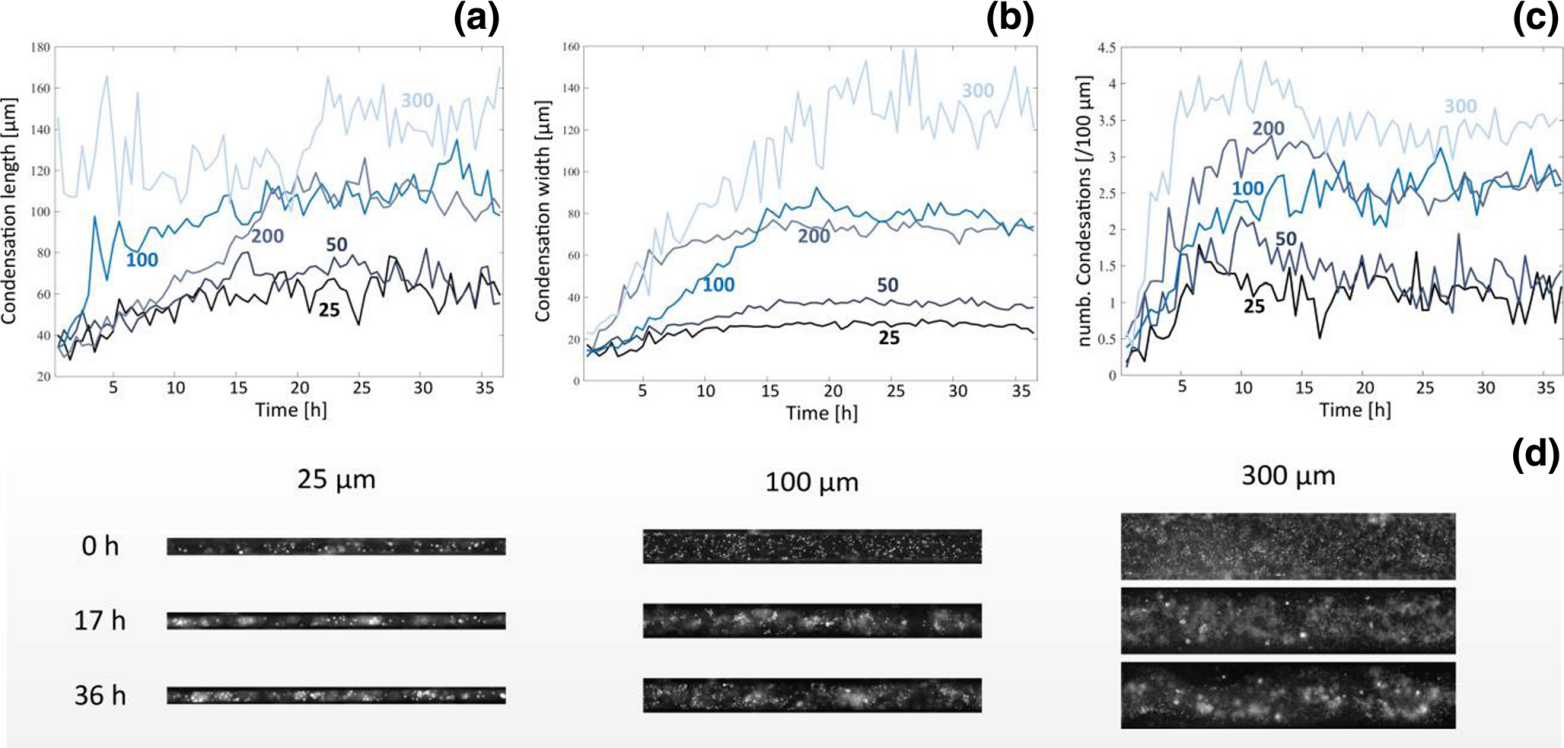
High content live imaging analysis of mesenchymal condensations. Evolution of (**a**) length, (**b**) width and (**c**) number of condensations measured over the 36 h of the live imaging for different groove sizes (25–300 μm). (**d**) Snapshot of individual grooves at representative time points

Combining the microtopography of the substrate with high content imaging analysis allowed investigating the role of geometric constraint on the development of mesenchymal condensations. We live tracked the establishment of condensations in limb bud ESPCs over 36 h (Fig. 4a-d). From this analysis we identified three different constraint regimes provided by small (25 and 50 μm), medium (100 and 200 μm) and large grooves (300 μm). Cells confined in small grooves showed a limited increase in the number and size of condensations. Indeed, condensation number increased to 1.2 and 1.3 per 100 μm while their length, which is the unconstrained dimension, increased only by 49% and 56% over the 36 h for 25 μm and 50 μm grooves respectively, indicating that the strong geometric constraint imposed by this topography highly limited the establishment of the condensations. In contrast, for intermediate size grooves (100 and 200 μm), condensations significantly grew in numbers and size. The number of condensations reached 2.6 and 2.4 per 100 μm length, while their length increased by 192% and 190% for 100 μm and 200 μm grooves respectively. The number of condensations stabilized at around 5 h while their size stabilized at around 17 h. Large groove size (300 μm) showed condensation numbers increasing to 3.6 per 100 μm length while their length remained largely constant throughout the experiment and was analogous to the length on medium sized grooves.

In this limited-to-none confinement setting, the condensations grew in width until they reached an almost unitary aspect ratio at 21 h (AR = 1.2). On the other hand, small grooves induced elongated condensations with aspect ratios greater than 2, while medium grooves showed intermediate aspect ratio values (AR ≈ 1.5). The constrained dimensions of the condensations, their width, also reached a steady-state value within 15 h for the small and medium grooves while the large grooves steadied at 21 h. The width was characteristic of the constraint regime. Small grooves had a 20–35 μm width, medium grooves a 70–80 μm width and large grooves a 125 μm width. This data overall indicates that microtopography does impose constraints on mesenchymal condensation which fall within three separate regimes. Small grooves tightly confined the establishment of condensations, limiting their length as well as their width, while medium grooves only limited the width of condensations without affecting length and the large grooves did not impose perceivable geometric constraints.

**Fig. 5:**
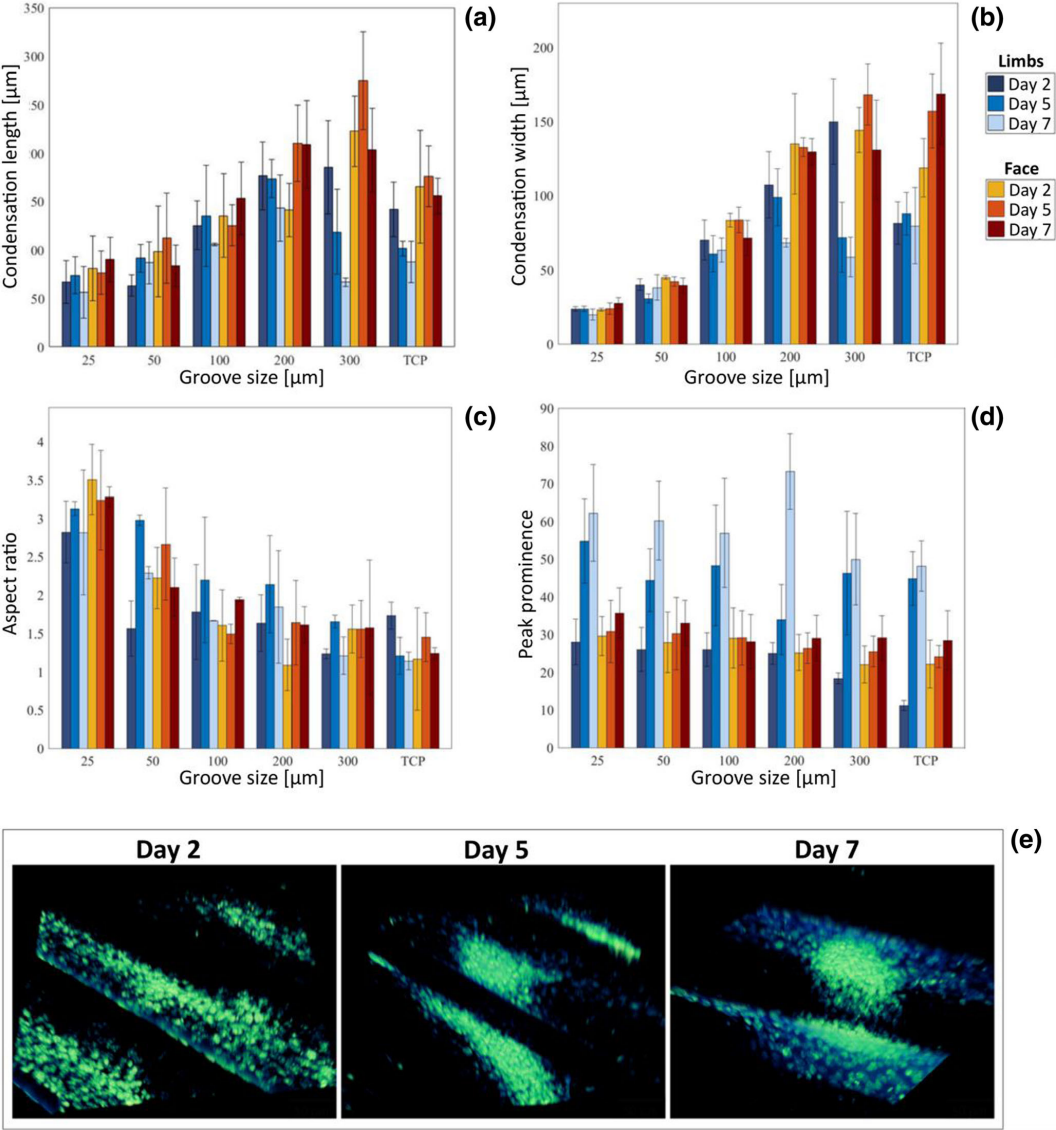
Long-term analysis of mesenchymal condensations. **a** Mesenchymal condensation width, (**b**) length, (**c**) aspect ratio and (**d**) peak prominence of limb buds (blue) and face (red) ESPCs at 2, 5, 7 days. **e** Confocal images of condensations at 2, 5 and 7 days

This view was confirmed in the analysis of long-term condensation maturation performed on fixed and stained samples at 2, 5 and 7 days (Fig. 5a-c). The length, width and overall aspect ratio for both face and limb bud condensations did not increase past day 2, agreeing with the live imaging observation that following 17 h the condensations have been established.

**Fig. 6:**
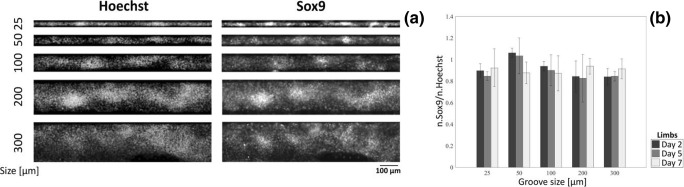
Lineage commitment of mesenchymal condensations. **a** Hoechst and Sox9 co-staining of ESPCs after 2 days in culture, for different groove sizes (25–300 μm). **b** Ratio of Sox9 + ve nuclei to total nuclei within mesenchymal condensations

A markedly different behavior instead was observed for peak prominence, a measure of the density of cells within the condensation (Fig. 5d). Limb bud condensations displayed a rapid increase of peak prominence between day 2 and day 5, which was further sustained until day 7. This increase in cell compaction was supported by confocal imaging displaying an increased cell density as time progressed (Fig. 5e) and suggests a continued proliferation within the condensation, indicating a more mature state. Face condensations displayed a similar, but slower, increase in prominence between 2 and 7 days (Fig. 5d), which also suggest progression towards maturation and well agrees with independent reports of a slower maturation kinetics for face vs limb condensations. To further support the maturation of condensations we identified that all cells within limb condensations, for all groove types were Sox9 positive (Fig. 6a-b), a marker for chondrogenic differentiation, starting from at least day 2.

## Conclusion

The establishment of mesenchymal condensations is a crucial first step in the differentiation of a wide range of embryonic organs including bone, cartilage, somites and kidneys. In this process, cells aggregate to form tightly packed groups which predict the final size and shape of the organ. Traditionally, high density micromass cultures of pre-chondrogenic mesenchyme have been used to mimic the *in vivo* condensation process. Relevant to tissue engineered bone and cartilage, micromass experiments have suggested that the ultimate shape and size of skeletal elements can be modulated by cell-cell or cell-matrix interactions within 3D spatial constraints. Therefore, we established an approach for the rapid assessment of the role of geometrical constraints during the establishment and maturation of micromass condensations. In the long term, our approach will be broadly applicable to testing and modifying multiple parameters in organogenesis.

First, it is crucial to have a standardized quantitative measure of the relevant cellular descriptors. On the basis of shape changes extracted from the segmentation of conventional brightfield micromass images of face and limb bud cells, we designed a 3D PDMS platform to study the effects of uniaxial constriction on condensation formation. We chose ‘linearisation’ via geometrical constraint of these cellular condensations in order to allow simplification and expansion of existing mathematical models of mesenchymal condensation. To improve the biocompatibility of PDMS substrates and to maintain long-term cultures, we optimised the combination of adhesive proteins and antifouling treatments.

High content imaging analysis of this platform, alongside confirmatory confocal and molecular analysis revealed two distinct regimes of uniaxial constriction on the establishment of mesenchymal condensations. For constriction between 25 μm and 50 μm, the constraint affected the elongation of the condensation along the free dimension, while for constrictions between 100 μm and 200 μm elongation along the free dimension was unconstrained, leading to increased aspect ratios. At 300 μm there were no constrictions as the condensations evolved isotropically with an aspect ratio of 1, analogous to what observed in unconstrained systems. Regardless of the constriction regimes all condensations reached their final dimensions by 17 h post-seeding, then until 7d they increased in cell density without significantly altering their size. Throughout this process the cells present within limb condensations were committed to chondrogenic differentiation. This phenotypic evolution leads us to hypothesise the presence of two distinct stages in the development of mesenchymal condensations: an initiation phase during the 0-17 h period where cells are locally recruited to establish a condensation, followed by a maturation phase lasting at least until day 7 during which cells within the condensation continue to proliferate, increasing its density rather than its size. This hypothesis requires further confirmation in a study combining high content analysis with molecular and cell biology investigations, aimed at identifying the biological processes underlying the observed evolution of phenotype. Overall, these findings identify that the established 3D model is suitable to investigate emergent phenotypes originating from complex multiscale cellular interactions within mesenchymal microenvironments. Indeed, refinement of our approach will allow us to test several conflicting models in the field, where cells are either brought together by attractive forces to form a compacted aggregate, or, alternatively, short range repulsive forces define the size and density of the aggregates. These two models may not actually be in conflict, but may instead represent two phases in the developmental process. The guidelines extracted from the refinement of our approach can provide design principles for scaffold tailored to the specific stages of mesenchymal condensation that we have here identified, allowing developing strategies to dynamically support skeletal morphogenesis.

## Methods

### Micromass image segmentation

Brightfield images of face and limb buds cells (Fig. 1a) at 20 days in normal micromass culture were analysed with Matlab® (2017b) to extract condensation shape descriptors. Images were first converted to grayscale (Fig. 1b), preprocessed to enhance contrast by contrast-limited adaptive histogram equalization (CLAHE) and low-pass filtered to remove constant power additive noise before being binarized (Fig. 1c) with Otsu’s method. Morphological opening was performed to remove any small white noises in the image, and morphological closing to remove any small holes in the object. All connected components that had fewer than 24 pixels were removed and structures that were lighter than their surroundings and connected to the image border were suppressed. The images were segmented by a watershed transformation (Fig. 1d) and a distance transform was used as segmentation function to split out the regions. Watershed transform is known for its tendency to “oversegment” an image because each local minimum, no matter how small, becomes a “catchment basin”. For this reason we (i) filtered out tiny local minima using *imextendedmin* (ii) modified the distance transform so that no minima occured at the filtered-out locations. This procedure is called “minima imposition” and was implemented via the function *imimposemin*.

With watershed segmentation, single condensations were identified. The properties of the image regions were directly or indirectly calculated through the *regionprops* function (Table 1).

Linear Discriminant Analysis (LDA) between face and limb buds condensation shape descriptors was performed (Fig. 1e). Shape descriptors were extracted from experiments with three technical repeats with three biological replicate for each one. The obtained values were standardized for all of the predictors (shape descriptors) and the regression model was fitted with the *fitcdiscr* function of Matlab®.

### 3D microwells fabrication

Polydimethylsiloxane (PDMS) grooves (25, 50,100, 200, 300 μm width) were fabricated by soft lithography. Negative photo resist SU-82100 was deposited onto clean and dried silicon wafers with a thickness of 100 μm and patterned by exposure to UV light through a transparency photomask. The stamp mold was silanized with vapour phase deposition of trichloro (1H, 1H, 2H, 2H-perfluorooctyl) silane (Sigma-Aldrich) to allow for an easy peel-off of the PDMS. PDMS prepolymer base and curing agent from a Sylgard 184 kit were mixed in a 10:1 ratio. The bubbles were removed by degassing in a vacuum desiccator. The PDMS was spin-coated on the patterned silicon wafer (400 rpm for 40s with acceleration of 1000 rpm/s) and cured at 95 °C for 10 min on a hotplate. After cooling, PDMS was peeled off the mold and the stamps were cut in 6 mm diameter discs and stored in 100% ethanol.

### Surface functionalization

To chemically functionalize the surface of the material, different coating strategies were tested. First, the PDMS microstructures were rendered hydrophilic by exposing them to oxygen plasma treatment (3 min 70 W at 0.4mBar, with an *O*_2_ flow rate of 20 sccm; Diener Zepto-W6). Thereafter, the substrates were coated with Poly-D-lysine (PDL, Sigma-Aldrich, 1 mg/10 ml in sterile water), Poly-L-lysine (PLL, Sigma-Aldrich, 1 mg/10 ml in sterile water), Gelatin (0.1% in PBS), Collagen type 1 (Roche, #111791179001, 5 mg/ml in PBS), or Fibronectin (5μg/cm^2^ in PBS, Sigma-Aldrich). The solutions were incubated for 2 h, the excess of liquid was aspirated, and the PDMS discs were left to become dry overnight. Coatings were washed twice with PBS prior to cell plating.

A coating strategy with (3-aminopropyl)triethoxy silane (APTES, Sigma-Aldrich) to immobilize adhesive proteins (Fibronectin) was developed (Fig. 2a). The PDMS microstructures were rendered hydrophilic by exposing them to oxygen plasma treatment (3 min 70 W at 0.4mBar, with an *O*_2_ flow rate of 20 sccm; Diener Zepto-W6). The substrates were coated with APTES solution (5% in 100% ethanol (*v*/v)) for 2 h at RT, washed three times with 100% ethanol and washed three times with PBS. Each mould was then coated with 5μg/cm^2^ fibronectin in PBS for 2 h at RT and washed two times with PBS.

### Micromass culture

Animal husbandry was carried out in accordance with King’s College London ethical guidelines and UK Home Office Licence P8D5E2773. Facial prominences (including the frontonasal process, maxilla and mandible) and limb buds were collected from E12.5 embryos (CD1 strain). Pooled facial and limb tissues were triturated. Cells were then enzymatically dissociated in a water bath using Dispase (Roche, 1unit/ml in 10% FBS) for 1 h 15 min shaking (70 rpm) at 37 °C. Gentle vortexing was carried out every 15mins. Dispase was neutralised with an equal volume of micromass media (50:50 DMEM:F12 (Sigma), 10% FBS (Gibco), 1X ABAM (Sigma), 2 mM L-glutamine (Sigma), 5 mM Beta-glycerophosphate (Sigma) and 0.1mg/ml Ascorbic acid (Sigma)) and passed through a 40 μm cell strainer (Flowmi, Belart). Limb and facial prominence plating densities were 200,000 and 1,000,000 cells per well respectively (in a 20ul droplet). Droplets were incubated for 1-2 h in 37 °C with 5% CO_2_. Humidity was maintained by placing PBS in the inter-well space of the plate. Wells were inspected for cell attachment then media was added and cells were incubated for the stated time.

### Staining

Cells were fixed using 4% PFA for 10 mins at room temperature. A 5 min permeabilisation step was performed (0.5% TritonX-100 (Sigma) in 1X PBS) at room temperature. Then, blocking solution was applied (3% BSA (Sigma) in 1X PBS, 0.01% Tween20 (Sigma)) for 1-2 h at room temperature. Primary anti-SOX9 (Millipore, 1:500) in blocking solution was incubated overnight at 4 °C. Samples were washed three times (1% BSA, 0.01%Tween20) and then incubated in 1:500 far red anti-Rabbit (Alexafluor), 20μg/ml Hoechst 33342 (Sigma) and 1:500 Phalloidin-488 (Life Technologies) in blocking solution for 1-2 h. Samples were washed three times and then plates were imaged using a Leica DMi8 or Nikon Spinning Disc Confocal.

### Live imaging

Micromass media was supplemented with 0.02μg/ml Hoescht 33342 (Sigma) as a DNA stain during live imaging. Cells were maintained at 37 °C with 5%CO_2_ in an environment chamber (Solent scientific). Brightfield and Hoescht fluorescence images were collected every 30 mins for a total of 36 h using a Leica DMi8.

### Stained images and high content live imaging analysis

To quantify condensation characteristics, images of Hoechst nuclear staining at 36 h, 2, 5 and 7 days of culture were analysed using Matlab® (2017b). Condensations were identified with fluorescence intensity analysis. First, single grooves were selected (Fig. 3a). Mesenchymal condensation length is the size of the condensation along the major axis of the groove and is given by full width at half maximum (FWHM) for peaks of Hoechst fluorescence averaged along the minor axis of the groove (Fig. 3c). Peak prominences were extracted as well (with the function *findpeaks*) to obtain the peak intensity values of the single condensations. For each peak identified, mesenchymal condensation width is given by FWHM for peaks of Hoechst fluorescence averaged along the major axis of the groove (Fig. 3b). Aspect ratio was calculated as ratio between condensation length and width (except for 300 μm grooves and tissue culture plate (TCP) where, due to the fact that condensation sizes are not confined, it is calculated as ratio between the longest and the shortest size).

In order to observe the evolution of mesenchymal condensation parameters over 36 h within each groove, condensations were identified at the final timepoint and tracked backwards to their initial establishment.

All parameters were measured per condensation and then averaged over a range of replicate condensation.
